# Efficacy of Essential Oils in Pain: A Systematic Review and Meta-Analysis of Preclinical Evidence

**DOI:** 10.3389/fphar.2021.640128

**Published:** 2021-03-01

**Authors:** Damiana Scuteri, Kengo Hamamura, Tsukasa Sakurada, Chizuko Watanabe, Shinobu Sakurada, Luigi Antonio Morrone, Laura Rombolà, Paolo Tonin, Giacinto Bagetta, Maria Tiziana Corasaniti

**Affiliations:** ^1^ Pharmacotechnology Documentation and Transfer Unit, Section of Preclinical and Translational Pharmacology, Department of Pharmacy, Health and Nutritional Sciences, University of Calabria, Rende, Italy; ^2^ Regional Center for Serious Brain Injuries, S. Anna Institute, Crotone, Italy; ^3^ Laboratory of Chemical Pharmacology, Faculty of Pharmaceutical Sciences, Daiichi University of Pharmacy, Fukuoka, Japan; ^4^ Center for Supporting Pharmaceutical Education, Faculty of Pharmaceutical Sciences, Daiichi University of Pharmacy, Fukuoka, Japan; ^5^ Department of Physiology and Anatomy, Faculty of Pharmaceutical Sciences, Tohoku Medical and Pharmaceutical University, Sendai, Japan; ^6^ Section of Preclinical and Translational Pharmacology, Department of Pharmacy, Health and Nutritional Sciences, University of Calabria, Rende, Italy; ^7^ Department of Health Sciences, University “Magna Graecia” of Catanzaro, Catanzaro, Italy; ^8^ School of Hospital Pharmacy, University “Magna Graecia” of Catanzaro, Catanzaro, Italy

**Keywords:** essential oils, pain models, inflammatory pain, neuropathic pain, chronic pain, systematic review, meta-analysis

## Abstract

**Background:** The demand for essential oils (EOs) has been steadily growing over the years. This is mirrored by a substantial increase in research concerned with EOs also in the field of inflammatory and neuropathic pain. The purpose of this present systematic review and meta-analysis is to investigate the preclinical evidence in favor of the working hypothesis of the analgesic properties of EOs, elucidating whether there is a consistent rational basis for translation into clinical settings.

**Methods:** A literature search has been conducted on databases relevant for medical scientific literature, i.e., PubMed/MEDLINE, Scopus, and Web of Science from database inception until November 2, 2020, following the PRISMA (Preferred Reporting Items for Systematic reviews and Meta-Analyses) criteria for systematic reviews and meta-analyses.

**Results:** The search was conducted in order to answer the following PICOS (participants/population, interventions, comparisons, outcomes, and study design) question: are EOs efficacious in reducing acute nociceptive pain and/or neuropathic pain in mice experimental models? The search retrieved 2,491 records, leaving 954 studies to screen after the removal of duplicates. The title and abstract of all 954 studies were screened, which left 127 records to evaluate in full text. Of these, 30 articles were eligible for inclusion.

**Conclusion:** Most studies (27) assessed the analgesic properties of EOs on acute nociceptive pain models, e.g. the acetic acid writhings test, the formalin test, and the hot plate test. Unfortunately, efficacy in neuropathic pain models, which are a more suitable model for human conditions of chronic pain, had fewer results (only three studies). Moreover, some methodologies raised concerns in terms of the risk of bias. Therefore, EOs with proven efficacy in both types of pain were corroborated by methodologically consistent studies, like the EO of bergamot, which should be studied in clinical trials to enhance the translational impact of preclinical modeling on clinical pain research.

## 1 Introduction

### 1.1 Rationale

Essential oils (EOs) containing components in exact proportion contributing synergically to the whole plant effect, have been used in traditional medicine for centuries since *The Divine Farmer’s Materia Medica*, the first text of Chinese Traditional Medicine, representing a form of combinatorial medicine (Li and Weng, 2017). The search for natural and green products is constantly increasing the use of essential oils and the demand for these products from developing countries. There has been a remarkable increase in the import of EOs by the European market from 2011–2018 (Eurostat) and it is estimated that the demand for essential oils in the global market will grow by 7.5% from 2020 to 2027 (GVR, 2020). These data are mirrored by the steady increase of research on EOs that pave the way for the development of these products.

**FIGURE 1:**
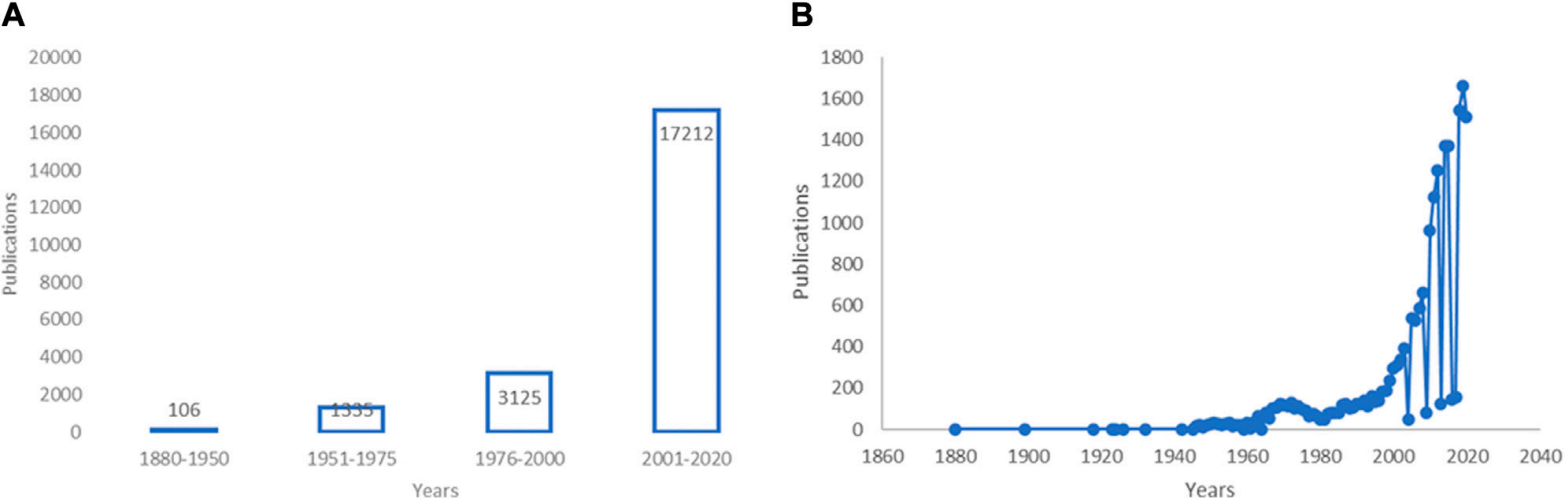
Research in the field of essential oils (EOs) over the years.**(A,B)** Increase of research in the field of essential oils (EOs). **(A)** A PubMed advanced search using the key word “essential oils” combined with the dates of publication from 1880 to present through the Boolean operator AND has retrieved an increase from 106 to 17,212 (date of last search November 19, 2020) of results. The first interval “1880–1950” is wider because no great amount of research in this field has been detected up until the 1950s. **(B)** Data are presented per year of publication based on search query “essential oils” (date of last search November 25, 2020). Modified from (Scuteri et al., 2017a).

Identifying the year 1880 as this field emerged (Wood and Reichut, 1880), we found a remarkable increase in publications concerned with EOs up to 2020 (Figures 1A,B) (see also (Scuteri et al., 2017a)). EOs have shown several beneficial properties, many of which concern the treatment of neurologic diseases, mood disturbances, and pain. Modulation of the γ-aminobutyric acid (GABA) neurotransmission and blockade of neuronal voltage-gated sodium channels (Na + channels) as well as activity on serotonergic neurotransmission are proposed as mechanisms involved in the action of EOs endowed with anxiolytic and anti-nociceptive properties like bergamot essential oil (BEO) (Rombolà et al., 2017; Scuteri et al., 2018a; Scuteri et al., 2019a; Scuteri et al., 2019b; Rombolà et al., 2019; Rombola et al., 2020), lavender essential oil (LEO) (Lopez et al., 2017), and melissa (lemon balm) (Abuhamdah et al., 2008; Awad et al., 2009). The cholinergic system is targeted by extracts of plants as sage (Perry et al., 2000; Savelev et al., 2003; Savelev et al., 2004), ginkgo (Stein et al., 2015; Zhang et al., 2018), and lemon balm (Dastmalchi et al., 2009; Guginski et al., 2009), showing therapeutic potential for diseases like dementia. The gathered evidence shows the potential benefits of EOs in the treatment of pain in fragile patients for whom several drugs can be more harmful, e.g. in aging or chronic neurologic diseases such as dementia (Achterberg et al., 2020). Pain is associated with mood disturbances (Evans, 1987; Husebo et al., 2011) influenced by aging (Hamm and Knisely, 1985; Scuteri et al., 2020a) and neuropathology (Scherder et al., 2003) and its treatment represents a field of strong inappropriateness in patients suffering from Alzheimer’s disease. (Scuteri et al., 2017b; Scuteri et al., 2018b; Achterberg et al., 2020; Scuteri et al., 2020f). Therefore, aromatherapy represents a fundamental tool for the safer handling of pain.

Despite a large amount of continuously growing research on EOs, a real translation of aromatherapy into clinical settings and the treatment of pain has not occurred. Research efforts have aimed to discover the mechanisms at the root of the analgesic activity of EOs, often focusing on the single components commonly present in different plant oils e.g., linalool, limonene, pinene, eugenol, and cinnamal. For instance, linalool, limonene, and pinene contribute to the anxiolytic and antidepressant properties of some EOs (see (Lizarraga-Valderrama, 2020)). In particular, some natural components of plants have been suggested as possible candidates for an analgesic action in neuropathic pain (Quintans et al., 2014). However, the strongest effect of EOs is due to the whole phytocomplex made up of various plant components that need to be present in a precise ratio to exert the so called *entourage* effect (Ribeiro, 2018). Definite combination of the constituents of EOs is necessary, but further studies are needed to highlight the exact active composition for each EO. The EOs of the species Citrus contain volatile components (85–99%), most abundantly terpenoids, and a non-volatile fraction including coumarins i.e. bergapten inducing phototoxicity (Zaynoun et al., 1977). Thus, the EO of bergamot has been deprived of bergapten (Bagetta et al., 2010), but is still endowed with its characteristics. The EO of bergamot can modulate the synaptic level of glutamate and this occurs when it is used as a bergapten-free fraction (Morrone et al., 2007). Hence, a mixture of monoterpene hydrocarbons within the volatile fraction may be responsible for bergamot analgesic activity since glutamate is significantly involved in the pain descending pathway due to metabotropic glutamate receptors mGluR7 and mGluR8 (Boccella et al., 2020). The novel phytocannabinoid cannabidihexol, with the terpenophenolic core of cannabidiol and Δ9-tetrahydrocannabinol, has proven to significantly reduce the late phase of the formalin test at low doses in C57BL/6J mice (Linciano et al., 2020). Cannabidiol oil has been demonstrated to reduce traumatic brain injury-induced allodynia (Belardo et al., 2019). Certain EOs have been proven to have enhanced efficacy if combined: e.g., peppermint and caraway oil are significantly effective on post-inflammatory visceral hyperalgesia only when used in combination (Adam et al., 2006). Likewise, the route of administration and the time of exposure can influence the effects of EOs (Scuteri et al., 2018a; Koyama and Heinbockel, 2020). Moreover, some EOs are efficacious in a preclinical setting (Sarmento-Neto et al., 2015), but often only in a definite model of pain, usually acute e.g. the acetic acid-induced writhings, that does not find a significant counterpart in clinic. Furthermore, EOs are often administered as *gavage* or for inhalation not always allowing an exact determination of the dose.

Clinical trials in aromatherapy are few, small and methodologically limited, hence it is not always possible to draw rigorous conclusions, particularly in dementia. As recently demonstrated in a Cochrane systematic review by Ball et al. (2020), the design, reporting and consistency of outcome measurement have been identified as the weakest points and need to be improved in the future. Thus, despite accumulating preclinical and clinical evidence for EOs (Scuteri et al., 2020d) and nutraceuticals (Scuteri et al., 2020e) in lots of forms and supplements, which have been studied in several neurodegenerative conditions, a sound rationale for their clinical use, especially in treating chronic pain (Lakhan et al., 2016), has not yet emerged.

## 2 Methods

### 2.1 Objectives

The present systematic review and meta-analysis aimed to verify the working hypothesis that EOs have analgesic properties by investigating preclinical evidence in favor of the latter, to understand whether there is a consistent rational basis for clinical translation. For this purpose, the objective was to assess the efficacy of EOs in preclinical models of both nociceptive and neuropathic pain through the PRISMA (Preferred Reporting Items for Systematic reviews and Meta-Analyses) (Liberati et al., 2009; Moher et al., 2009) criteria for systematic reviews and meta-analyses. The systematic review and meta-analysis focuses on the following PICOS (participants/population, interventions, comparisons, outcomes, and study design) question: are EOs efficacious in reducing acute nociceptive pain and/or neuropathic pain in mice experimental models? In particular, this work aimed at evaluating:

- analgesic effectiveness (outcome);
- of EOs with a known composition (interventions), and not single components or extracts, administered intraperitoneally (i.p.) or subcutaneously (s.c.) to allow determination of the exact dose and reproducibility;
- in male mice subjected to nociceptive or neuropathic pain models (participants/population);
- with respect to providing a vehicle or other treatments (comparators);
- in studies designed according to legislation to minimize the suffering of animals (study design).

To the best of our knowledge, this is the first meta-analysis of preclinical studies on the analgesic effects of EOs interventions in models of both nociceptive and neuropathic pain.

### 2.2 Protocol

The search strategy and extraction of data to answer to PICOS question followed the PRISMA (Liberati et al., 2009; Moher et al., 2009) criteria. Due to the nature of preclinical animal intervention systematic review and meta-analysis, the latter aims at investigating the consistency of the body of evidence for clinical translation without an outcome of clear human relevance. For this reason, it has not been registered in the International prospective register of systematic reviews PROSPERO. However, statistically analyzing basic research independent studies testing the same hypothesis with comparable parameters can: determine its consistency allowing to study that phenomenon in a larger sample surmounting the issues concerned with small sample sizes; correct confounders; improve reproducibility (Editorial, 2016). Thus, a systematic review and meta-analysis is fundamental to establish a real possible clinical translation of a preclinically studied effect since it can highlight whether it has been consistently proven with the most reliable human disease modelled approach. Two independent researchers ran the search in agreement with the previously established protocol and inclusion and exclusion criteria, including double-checking the retrieved results, and any conflicts found by them were resolved by a third author.

### 2.3 Eligibility Criteria

#### 2.3.1 Inclusion Criteria

The analysis included studies assessing the antinociceptive effect of EOs, administered i. p. or s. c. to allow determination of the exact dose and reproducibility, with a known percentage of components on male mice subjected to nociceptive or neuropathic pain models. Compliance with animal welfare regulations was an inclusion criterion of the utmost importance. The studies included needed to be designed according to legislation to minimize animal suffering. Either acute nociceptive or neuropathic pain models are included. Independently of the model used, the outcome of the study had to be antinociception for eligibility.

#### 2.3.2 Exclusion Criteria

Studies on species different from mice or any strains and female gender were not eligible. The use of different species and genders would not allow comparison and the number of papers examining pain in non-rodent species is very small. Papers in which extracts or single plant components are used were excluded. Studies that did not consider ethics were excluded. Narrative or systematic reviews and meta-analysis, *in vitro* studies, abstracts and congress communications, proceedings, editorials, book chapters, and studies not published in English and not available in full text were not eligible.

### 2.4 Information Sources

A literature search was performed on PubMed/MEDLINE, Scopus, and Web of Science. Embase could not be searched as it was not freely/institutionally available. No restriction of publication date was applied and databases were searched for records matching the search strings used from their inception. The date of the last search was November 2, 2020. After the elimination of duplicate records, the first screening evaluated the title and abstract, and then the full text was assessed to define inclusion in qualitative and/or in quantitative synthesis.

### 2.5 Search Strategy

The following search terms and modifications were used as key words in combination: *essential oils, pain, animal pain models, antinociceptive activity, allodynia, Von Frey (‘s test), hyperalgesia, Hargreaves (‘test), hot plate, capsaicin test, formalin test, tail flick test, acetone test, complete Freund's adjuvant, streptozocin, chemotherapy(-induced), oxaliplatin, cisplatin, paclitaxel, docetaxel, vincristine, vinblastine, eribulin, bortezomib, thalidomide, neuropathy, mice.*

### 2.6 Data Collection Process

**FIGURE 2:**
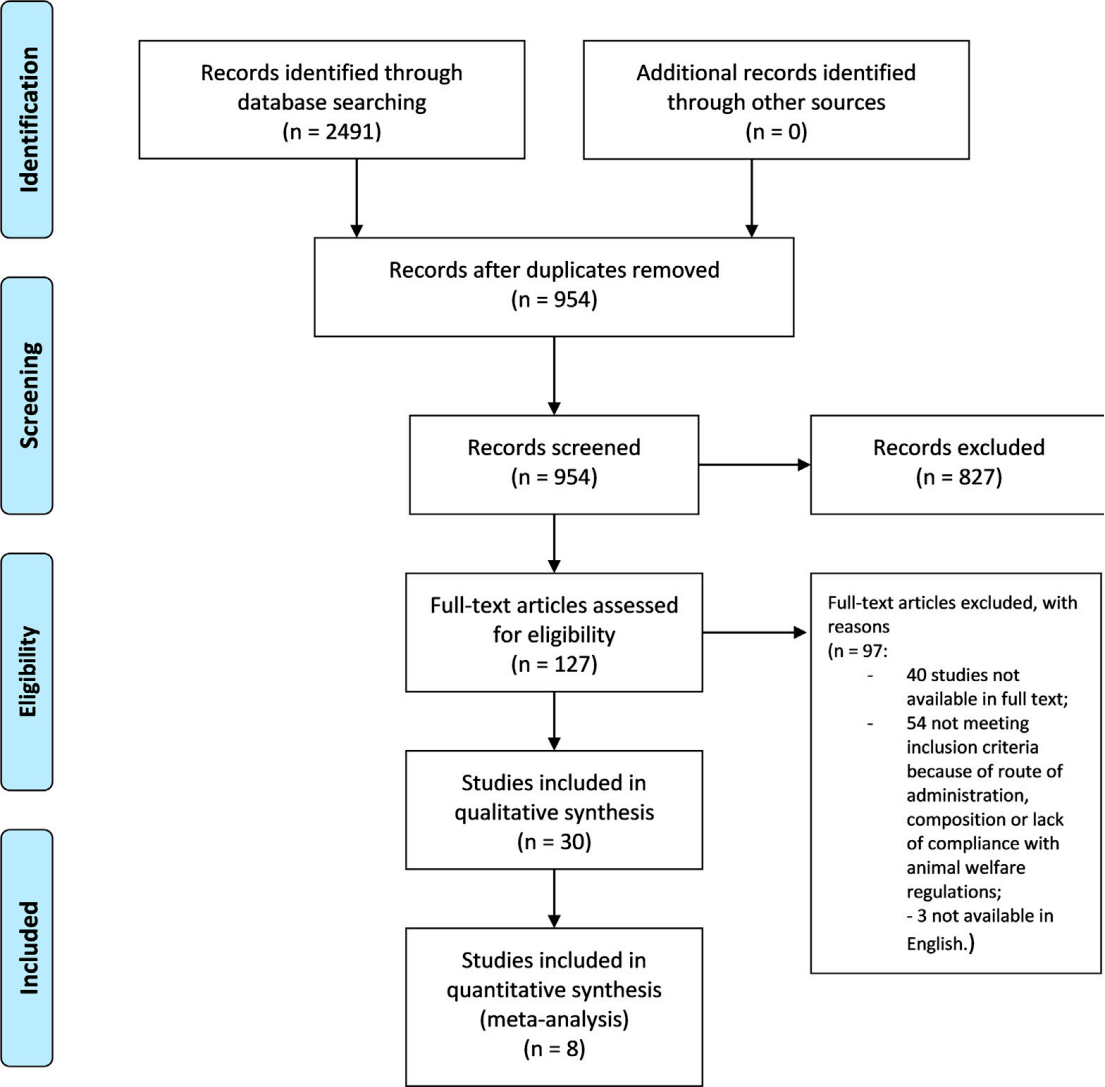
Literature search and screening of retrieved records. PRISMA flow diagram (Moher et al., 2009) of the selection process of the studies eligible for qualitative and quantitative synthesis.

The eligibility of the studies was assessed independently by two authors to minimize the risk of excluding relevant records. The references list of the articles was examined to extend and refine the search. A complete consensus was reached and no relevant conflicts were raised. The selection process is illustrated in the PRISMA flow diagram (Figure 2).

### 2.7 Synthesis, Risk of Bias, and Statistical Analysis

A systematic and narrative synthesis of the results, according to the Cochrane Consumers and Communication Review Group guidelines (Ryan, 2019 http://cccrg.cochrane.org, March 13, 2019 (accessed DATE).) was carried out. The risk of bias (internal validity) and the quality of the studies was assessed by two independent researchers through tools specific to preclinical animal studies like the Systematic Review Center for Laboratory Animal Experimentation (SYRCLE’s) risk of bias (RoB) tool (Hooijmans et al., 2014) and the Collaborative Approach to Meta-Analysis and Review of Animal Data from Experimental Studies (CAMARADES) checklist for study quality (Macleod et al., 2004). Any discrepancies were resolved through consensus or with the help of a third author.

Meta-analyses were conducted using Cochrane Review Manager 5.3 (RevMan5.3; Copenhagen: The Nordic Cochrane Center, The Cochrane Collaboration). A minimum of five articles per outcome measure was required according to the systematic review protocol for animal intervention studies by SYRCLE. When the tests included in articles were multiple and performed at different times and doses, only the most significant time point for pain development and progression in the specific model was considered for meta-analysis and only data related to the most efficacious dose were included. Studies expressing the analgesic outcome in a comparable way were included in the meta-analysis. Data available and comparable, but not expressed with the same measure of effect size as proportional reduction of outcome in treated animals relative to untreated controls were converted in mean and standard deviation to allow statistical comparison. Data not available and not extractable from graphs using digital ruler software, e.g., PlotDigitizer 2.6.9, were excluded from quantitative analysis. The Higgins I^2^ value was calculated to assess the heterogeneity of studies (Higgins and Thompson, 2002). Differences were presented as risk ratios (RR) including 95% confidence intervals (CI), using a random effect model (DerSimonian and Kacker, 2007) to manage the eventual heterogeneity of the studies and to assess intra- and inter-study variation. Publication bias was assessed through Egger’s linear regression test to measure funnel plot asymmetry, adjusted through the “trim and fill” method (Egger et al., 1997; Duval and Tweedie, 2000; Sterne and Egger, 2001).

## 3 Results

### 3.1 Selection Process and Data Collection

The search retrieved 2,491 results from databases and there were no results from additional searches. The records were screened for duplicates, leaving 954 studies. Title and abstract screening led to an initial exclusion of narrative or systematic reviews and meta-analysis, *in vitro* studies, abstracts and congress communications, proceedings, editorials, book chapters. This left 127 records in full text. Among these, two had to be excluded because the text was in Chinese (Li et al., 1991; Chen et al., 2011) and one was excluded because it was written in Spanish (Do Nascimento Silva et al., 2018). After full text screening, 30 studies were included in qualitative analysis: 40 studies were not available in full text and 54 were excluded because they did not meet inclusion criteria because of species used, route of administration, composition, or lack of compliance with animal welfare regulations. For instance, the study by Ali et al. (2012) in which the EO of Nepeta pogonosperma Jamzad et Assadi was proven to have significant efficacy at different doses in the tail-flick and formalin test in Wistar rats was therefore not eligible. Among the records included in qualitative analysis, eight were included in quantitative synthesis, reporting comparable outcomes and the exact number of animals used. The process of literature search and screening was illustrated in the PRISMA flow diagram (Moher et al., 2009) in Figure 2.

### 3.2 Qualitative Synthesis

The data obtained from the 46 studies included in the qualitative analysis were grouped according to Cochrane Consumers and Communication Review Group guidelines (Ryan, 2019 http://cccrg.cochrane.org, March 13, 2019 (accessed DATE).). These groups were based on the experimental pain model used in 1) EOs showing analgesia in nociceptive models, and 2) EOs with analgesic properties in neuropathic pain. The majority (27/30) of the studies used an acute nociceptive model. Studies providing a range and not an exact number of animals per group were not considered eligible for quantitative analysis. Studies expressing the analgesic outcome in a not comparable manner to the majority were excluded from the meta-analysis. The main characteristics of the studies with reference to the PICOS question are reported in Tables 1, 2.

**TABLE 1:** Main characteristics of the studies included showing the efficacy of EOs in nociceptive models.

Study	EO [most representative components]	Route of administration	Control	Mice strain	Pain model	Analgesic outcome and sample size	Design
Anaya-Eugenio et al. (2016)	Artemisia ludoviciana [7)-camphor (21%), γ-terpineol (18%), borneol (18%),terpine-4-ol (3.5%),1,8-cineole (3.4%),lavanderlactone (3.4%), isoborneol (2.4%),camphen-6-ol (2.3%), *trans*-sabinylacet-ate (2.2%),andbornylacetate (2.2%]	i.p	Saline	ICR mice	1) Hot-plate test and 2) formalin test	1) Dose-dependent antinociceptive action (*n* = 8); 2) effectiveness in the first phase at the highest dose and in dose-dependent manner in the second phase (*n* = 8)	Use of increasing logarithmic dose (1, 10, 17.7, 31.6, and 100 mg/kg) according to allometric scaling. Use of a References drug: morphine sulfate. Antagonism studies with naloxone, atropine sulfate, L-nitro arginine methyl ester (l-NAME), or glibenclamide. No sample power calculation, randomization or blinding, or conflict of interest statement
Andrade et al. (2012)	Myrcia pubiflora DC., myrtaceae [caryophyllene oxide (22.2%), mustakone (11.3%), 1,8-cineole (5.4%), and tricyclene (5.3%)]	i.p	Vehicle (saline + Tween − 80 0.2%)	Swiss mice	1) Acetic acid-induced writhing test, 2) formalin test and 3) hot plate test	All the doses (25, 50, and 100 mg/kg) were active in acetic acid (*n* = 8) and formalin test (*n* = 8) and no dose in hot plate test (*n* = 8)	Random housing. Experiments performed between 9am and 4pm. Use of References drug morphine. No conflict of interest statement
Bae et al. (2020)	Ocimum basilicum L. (basil, lamiaceae) [linalool (56.6%), eugenol (18.1%), 1,8-cineole (6.93%), γ-cadinene (6.43%), and β-pinene (1.84%)]	i.p	Control (0.9% saline); vehicle (almond oil)	C57BL/6 mice	1) Acetic acid-induced writhing test, 2) formalin test and 3) hargreaves’ test (*n* = 9)	EO (45 mg/kg) is effective in 1) acetic acid test (*n* = 8–10) and in 2) the second phase (*n* = 7–12), but only at a much higher dose (180 mg/kg) in 3) the hargreaves’ test (*n* = 9)	Positive and negative control drugs have been used: morphine, indomethacin, naloxone, 5′-guanidinonaltrindole, naltrindole, l-NAME; l-arginine, and glibenclamide-hippuric acid. Mice have been randomly divided into groups. No conflict of interest statement
Quintans et al. (2017)	Aristolochia trilobata L. [6-methyl-5-hepten-2-ylacetate (SA) (21.49 ± 0.43%), germacrene D (15.07 ± 0.23%), bicyclogermacrene (8.84 ± 0.45%), linalool (6.85 ± 0.42%), (E)-caryophyllene (5.58 ± 0.12%), (E)-β-ocimene (5.56 ± 0.067%) and *p*-cymene (4.68 ± 0.10%)]	i.p	Vehicle (saline +0.2% tween	Swiss mice	1) Acetic acid test and 2) formalin test	1) EO (25, 50 and 100 mg/kg) has exerted analgesia comparable to morphine and sulcatyl acetate (*n* = 8); 2) EO (25, 50 and 100 mg/kg) has resulted active in the second phase and only at the highest dose in the first phase (*n* = 8)	Mice have been randomly assigned to groups. Experiments have been carried out from 08:00 a.m. to 04:00 p.m. (during light period) and in a blind manner. Morphine, acetylsalicylic acid and sulcatyl acetatehave been used as positive controls. No conflict of interest statement
de Oliveira Júnior et al. (2017)	Croton conduplicatus kunth [(E)-caryophyllene (13.72%), and caryophyllene oxide (13.15%) and monoterpene camphor (8.25%)]	i.p	Vehicle (0.9% saline +10 µL of tween 80, 10 ml/kg)	Swiss mice	1) Acetic acid-induced writhing test, 2) formalin test and 3) hot plate test	EO has resulted active at all doses in acetic acid (*n* = 6) and formalin test (*n* = 6) and at dose of 50 mg/kg in the hot-plate test (*n* = 6)	All the studies have been carried out by the same observers. Morphine and indomethacin as positive control. Antagonism studies (glibenclamide, naloxone and atropine). Random housing. Declaration of no conflict of interest
de Oliveira et al. (2018)	Croton conduplicatus kunth [monoterpenes 1,8-cineole (21.42%) and *p*-cymene (12.41%) and sesquiterpenes spathulenol (15.47%) and caryophyllene oxide (12.15%)]	i.p	Vehicle	Swiss mice	1) Acetic acid-induced writhing test, 2) formalin test and 3) hot plate test	EO (25, 50 and 100 mg/kg) has resulted active in acetic acid (*n* = 6) and formalin test (*n* = 6) and no dose in hot plate test (*n* = 6)	All the studies have been carried out by the same observers. Morphine and indomethacin as positive control. Antagonism studies (naloxone, atropine and flumazenil). Random housing. Declaration of no conflict of interest
Guimaraes et al. (2009)	Eugenia candolleana DC. (myrtaceae) [β-elemene (35.87 ± 0.13%), δ-elemene (8.28 ± 0.02%), β-caryophyllene (8.15 ± 0.08%). viridiflorene (6.96 ± 0.05%) (Neves et al., 2017); leaves in serra da guia, poço Redondo-SE, Brazil (GPS: 9.58º47.05:S 37.52º24.11:W Guimaraes et al. (2009)]	i.p	Vehicle (saline +1 drop of Tween-80 0.2%)	Swiss mice	1) acetic acid-induced writhing test, 2) formalin test and 3) hot plate test	EO (25, 50, and 100 mg/kg) dose-dependently inhibits acetic acid (*n* = 10) and formalin-induced (*n* = 10), but not hot-plate (*n* = 10) behaviors	Experiments carried out between 09:00 h and 16:00 h. References drug was acetylsalicylic acid. Random housing. No conflict of interest statement
Hajhashemi et al. (2009)	Heracleum persicum [hexyl butyrate (56.5%), octyl acetate (16.5%), hexyl 2-methylbutanoate (5.2%), hexyl isobutyrate (3.4%) and n-octyl 2-methylbutyrate (1.5%)]	i.p	Vehicle (1% solution of tween	Swiss mice	Acetic acid-induced writhing test	EO (50–100 mg/kg) has reduced writhings of 66 and 73% respectively (*n* = 6), in comparison with the 80% reduction of indomethacin	Indomethacin has been used as positive control. No conflict of interest statement
Halder at al. (2012)	Eugenia caryophyllata (clove oil; myrtaceae) [eugeno (87.34%), eugenyl acetate (5.18%), and beta-caryophyllene (2.01%)]	i.p	0.9% saline	Swiss mice	1) Formalin test and 2) tail-flick test	1) EO (0.1 ml/kg) has reduced licking/biting time in the first and (0.025, 0.05, and 0.1 ml/kg) in the second phase (*n* = 6); 2) effect of clove oil (0.1 ml/kg) (*n* = 6) at 30 min of reduced tail-flick latency resulted reverted by naloxone. Though not significantly, the dose 0.025 ml/kg of clove oil, at 30 and 60 min have increased the mean tail-flick latency	Experiments performed at daytime between 09:30 and 15:30. Morphine and acetylsalicylic acid have been used as References drugs. Naloxone has been used in the tail-flick test. No conflict of interest statement
Khalid et al. (2011)	Zingiber zerumbet (L.) smith [zerumbone (36.12%), humulene (10.03%), humulene oxide I (4.08%), humulene oxide II (2.14%), caryophyllene oxide II (1.66%) and caryophyllene oxide I (1.43%), camphene (14.29%), borneol (4.78%), camphor (4.18%), eucalyptol (3.85%), α-pinene (3.71%), γ-terpinene (2.00%), β-phellandrene (1.63%), 1-terpen4-ol (1.44%), β-myrcene (1.22%) and linalool (1.06%), as previously described Sulaiman et al. (2010)]	i.p	Vehicle	ICR mice	Capsaicin, acetic acid, glutamate and phorbol 12-myristate 13-acetate (PMA)-induced nociception	EO (50, 100, 200, 300 mg/kg) has exerted significant dose-dependent inhibition of: abdominal writhings (*n* = 10); capsaicin-induced neurogenic nociception (*n* = 10) similar to that of acetylsalicylic acid and of capsazepine; glutamate-induced nociception (*n* = 10) and of PMA-induced nociception (*n* = 10), comparable to acetylsalicylic acid	Blinded, randomized and controlled design. Acetylsalicylic acid and capsazepine have been used as References drug. Antagonism studies with l-arginine, N^ὠ^-nitro-l-arginine, methylene blue and glibenclamide. No conflict of interest statement
Jahandar et al. (2018)	Pycnocycla bashagardiana (apiaceae) [myristicin (76.1%), E. β. Ocimene (4.1%), Z. β. Ocimene (3.8%) and β-eudesmol (2.9%)]	i.p	Vehicle	NMRI mice	1) Formalin test and 2) hot-plate test	1) EO (100, 200, and 400 mg/kg) has not shown antinociceptive activity in the formalin test (*n* = 6–8); 2) EO (50, 100, 200, and 400 mg/kg) has not shown antinociceptive activity in the hot-plate test	Morphine had been used as References drug. Declaration of no conflict of interest
Katsuyama et al. (2015)	Bergamot (citrus bergamia risso) [0.38% D-limonene, 70.26% linalyl acetate, 18.95% linalool, 0.62% γ-terpinene, and 0.03% β-pinene]	s.c	Jojoba wax and none	ddY mice	Formalin test	Significant dose-dependent inhibition of both phases by EO (2.5, 5, 10 µg) (*n* = 10)	All behavioral experiments have been carried out during the light period between 10:00 and 16:00. The animals have been tested in randomized order. Antagonism studies with naloxone hydrochloride and naloxone methiodide. No conflict of interest statement
Khodabakhsh et al. (2015)	Neroli (citrus aurantium L.) [linalool (28.5%), linalyl acetate (19.6%), nerolidol (9.1%), and E,E-farnesol (9.1%)]	i.p	Vehicle (sweet almond oil)	Wistar mice	1)acetic acid and 2) hot-plate test	1) Neroli (10 and 20 mg/kg) has significantly decreased the number of writhings (*n* = 8) and 2) has significantly increased latency time at dose of 40 mg/kg (*n* = 8)	Mice have been used only once and experiments have been conducted between 10.00 a.m. and 13 p.m. with normal room light. Diclofenac has been used as References drug and l-NAME as enhancer. No References to randomization of mice but only for the experimental part concerned with rats. No conflict of interest statement.
Lee et al. (2019)	Eucalyptus [(aromarant co. Ltd., rottingen, Germany); 1,8-cineole (61.46%), limonene (13.68%), ρ-cymene (8.55%), γ-terpinene (5.87%), α-pinene (4.95%), and α-phellandrene (1.09%) Jun et al. (2013)]	i.p	Control 0.9% saline and vehicle (almond oil)	C57BL/6 mice	1) Formalin test, 2) acetic acid test and 3) hargreaves’ test	1) EO (11.5, 22.5, 45 mg/kg) has significantly reduced licking time in the second phase (*n* = 7–12); 2) dose-dependent reduction of the number of writhes (*n* = 8–10); 3) no significant effect (*n* = 10)	Antagonism studies with the k-opioid antagonist 5′-guanidinonaltrindole, the δ-opioid antagonist naltrindole and the µ-opioid antagonist naloxone (also + morphine). Indomethacin has been used as References drug for acetic acid test. Declaration of no conflict of interest
Lima et al. (2012)	Chrysopogon zizanioides L. (roberty, poaceae) [khusimol (19.57%), E-isovalencenol (13.24%), α-vetivone (5.25%), vetiselinenol (5.08%), α-cadinol (5.01%), α-vetivone (4.87%) and hydroxy-valencene (4.64%)]	i.p	Vehicle (distilled water with one drop of tween 80 0.2%)	Swiss mice	1) Formalin test, 2) acetic acid test and 3) hot-plate test	1) EO (50, and 100 mg/kg) has been effective in the second phase (*n* = 10); 2) EO (50, and 100 mg/kg) has produced antinociception similar to morphine (*n* = 10); 3) EO (25, 50, and 100 mg/kg) has not shown efficacy (*n* = 10)	Morphine and acetylsalicylic acid have been used as References drugs and naloxone for antagonism study. Random housing. No conflict of interest statement
Ximenes et al. (2013)	Croton adamantinus müll. Arg. [Methyl-eugenol (14.81%), 1,8-cineole (13.74%), bicyclogermacrene (8.06%) and β-caryophyllene (5.80%)]	i.p	Vehicle (cremophor 0.5%, 0.1 ml/10 g)	Swiss mice	1) Formalin test and 2) acetic acid test	1) EO (50 and 150 mg/kg) is effective in both phases, and in a comparable way to morphine in the second phase; 2) EO (50 and 150 mg/kg) is more effective than indomethacin. Unreported number of animals	For the acetic acid test, the observation has been conducted by a blind observer. Morphine and indomethacin have been used as References drugs. No conflict of interest statement
Miraghazadeh et al. (2015)	Zhumeria majdae rech. F. and wendelbo (lamiaceae) [linalool (63.4%) and camphor (27.5%)]	i.p	Vehicle (sweet almond oil)	NMRI mice	1) Acetic acid test; 2) hot-plate test	1) EO (5, 10, 20, 40 mg/kg) has produced dose-dependent analgesia (*n* = 5); 2) EO (5, 20, 40 mg/kg) has significantly prolonged latency time in dose-related manner (*n* = 5)	Experiments have been conducted between 10.00 a.m. and 13.00 p.m. and mice used only once. Diclofenac has been used as References drug. No conflict of interest statement
Mishra et al. (2010)	*Senecio* rufinervis D.C. (Asteraceae) [germacrene D (40.19%), β-pinene (12.23%), β-caryophyllene (6.21%) and β-longipinene (4.15%)]	i.p	Vehicle (2% v/v tween 80)	Wistar albino mice	1) Acetic acid test; 2) hot-plate test	EO (25, 50, 75 mg/kg) has produced significant and dose-dependent inhibition of writhes and also of thermal hyperalgesia at the doses of 50 and 75 mg/kg (*n* = 6)	Pentazocine has been used as positive control. No conflict of interest statement
Nogueira et al. (2015)	Croton cordiifolius baill. (Euphorbiaceae) [1,8-cineol (25.09%), α-phellandrene (15.43%), β-cymene (8.02%), spathulenol (6.68%) and β-caryophyllene (6.58%)]	i.p	Vehicle (polyethoxylated castor oil - cremophor)	Swiss mice	1) Acetic acid test; 2) formalin test; 3) capsaicin test; 4) glutamate test	EO (50 and 100 mg/kg) has proven analgesia in all tests (*n* = 8) apart from capsaicin, comparable to indomethacin in its highest dose (acetic acid test) and higher than morphine (formalin test; in the second phase only at the highest dose). In gluatamate test only the highest dose has been effective. This effect id independent on naloxone	Indomethacin and morphine have been used as positive control and naloxone as antagonist. Declaration of no conflict of interest
Park et al. (2015)	Chamaecyparis obtuse [(mg/L): Isoprene 293, α-pinene 8,754, camphene 3,294, β-pinene 2,940, δ-3-carene 42, myrcene 36,440, α-phellandrene 697, α-terpinene 10,262, d-limonene 50,135, γ-terpinene 23,361, cymene 2,862, terpinolene 10,137, linalool 1824, camphor 250, bornyl acetate 74,903, α-humulene 1,628, terpineol 4,788, cedrol 7,230]	i.p	Control (distilled water containing 0.5% DMSO instead of treatments)	C57BL/6J mice	1) Formalin test, 2) acetic acid test, 3) hot-plate test and	EO (5 and 10 mg/kg) is effective in 1) formalin test (only at the lowest dose in the first phase) (*n* = 7–8) and in the 2) acetic acid test (*n* = 7–8), not in the hot-plate test (*n* = 10)	Acetylsalicylic acid has been used as positive control. No conflict of interest statement
Queiroz et al. (2014)	Xylopia laevigata (annonaceae) [γ-muurolene (17.78%), δ-cadinene (12.23%), bicyclogermacrene (7.77%), α-copaene (7.17%), germacrene D (6.54%), (E)-caryophyllene (5.87%), γ-cadinene (4.72%), aromadendrene (4.66%), and γ-amorphene (4.39%)]	i.p	Vehicle (saline +2 drops of tween 80 0.2%)	Swiss albino mice	1) Acetic acid test; 2) formalin test	EO (12.5, 25, 50 mg/kg) has significantly reduced the 1) acetic acid-induced writhings-induced nociception (*n* = 8) and the 2) two phases of the formalin test (*n* = 8)	Mice have been used only once. Morphine has been used as References drug. Naloxone has been used as antagonist. Declaration of no conflict of interest
Sakurada et al. (2011)	Bergamot (citrus bergamia risso) [0.38% d-limonene, 70.26% linalyl acetate, 18.95% linalool, 0.62% γ-terpinene and 0.03% β-pinene]	s.c	Saline, jojoba wax and none	ddy mice	Capsaicin test	Dose-dependent inhibiton of nociceptive response by the EO (2.5, 5, 10, 20 μg/paw) significant only at the highest doses of 10 and 20 μg/paw (*n* = 10)	Mice have been tested in randomized order and behavioral experiments have been performed during the light period between 10:00 and 17:00h. Lidocaine hydrochloride monohydrate and morphine hydrochloride have been used as References drugs. Naloxone hydrochloride and methiodide have been used as antagonists. No conflict of interest statement
Sharif et al. (2020)	Tanacetum balsamita (compositae) [carvone (39.8%) and α-thujone (11.9%).].	i.p	Vehicle (sweet almond oil)	NMRI mice	Hot-plate test	EO produced anti-nociception only at the dose of 100, mg/kg) (*n* = 6–8)	Experiments have been conducted between 10.00 a.m. and 13.00 p.m. and mice used only once. Morphine has been used as positive control. No conflict of interest statement
Ulku Karabay–Yavasoglu et al. (2006)	Satureja thymbra L. (lamiaceae) [γ-terpinene (40.99%), carvacrol (17.50%), thymol (13.19%), and P-cymene (12.73%), β-caryophyllene (3.15%), α-thujene (1.98%), and thymylmethylether (1.94%)]	i.p	Vehicle (2% tween 20)	Albino mice	Formalin test	Analgesic effect in the early (50 and 100 mg/kg) and in the late (25, 50, and 100 mg/kg) phases of the formalin test (*n* = 10)	Animals were used only once and humanly sacrificed at the end of the test. Morphine has been used as References drug and naloxone as antagonist. No conflict of interest statement
Venãncio et al. (2011)	Ocimum basilicum L. (lamiaceae) [linalool (76.13%), geraniol (11.16%), 1,8-cineol (6.66%)]	i.p	Vehicle (saline + tween 80 0.2%)	Swiss mice	Orofacial formalin, glutamate and capsaicin-induced nociception	EO (50, 100, 200 mg/kg) is effective in all the doses at the highest doses (*n* = 8)	Mice have been used once in the study. Nociception tests have been conducted by the same observer. Morphine hydrochloride and lidocaine have been used as References drugs. No conflict of interest statement
Zarei et al. (2018)	Inula britannica L. (asteraceae) [viridiflorol (7.17%–8.20%) and himachalol (3.45%–8.71%) followed by 6,10,14-trimethyl-2-pentadecanone (5.43%–2.95%), 13-tetradecanolide (3.93%–4.87%) and 3-methyl-4-propyl-2,5-furandione (4.06%–0.29%) Todorova et al. (2017)]. Flowers of inula britannica collected from the slopes of mount alvand, hamadan (34°47′59.99″N, 48°30′59.99″E	i.p	Control (saline)	Swiss albino mice	1) tail-flick test; 2) acetic acid test; 3) formalin test and 4) glutamate-test	EO (25, 50, and 100 mg/kg) (*n* = 5) for all tests. 1) the highest dose has resulted effective; 2) the doses of 50 and 100 mg/kg have been effective; 3) the highest dose is comparable to morphine; 4) only the highest dose is significantly active	Experiments have been carried out between 8 a.m. and 12 p.m. Morphine, naloxone, l-Arginine, methylene blue, glibenclamide, naltrindole, nor-binaltorphimine and naloxonazine have been used as positive and negative controls. Declaration of no conflict of interest
Zendehdel et al. (2015)	Bunium persicum (boiss.) [germacrene-d (22.1–24.1%) and E-caryophyllene (26.6–38%) Sofi et al. (2009)]. Samples of the plant identified at the division of pharmacognosy, faculty of pharmacy, tehran university of medical sciences, Iran	i.p	Control (Tween-80 (0.5%)	Albino NMRI mice	Acetic acid test	EO (0.001, 0.01, 0.05, 0.1, 0.5 and 1%; 10 ml/kg) (*n* = 7). The EO 0.01% has significantly reduced contortions (90.7% vs 38.13% of indomethacin) and this effect has been inhibited by naloxone and reduced by chlorpheniramine and cimetidine	Experiments have been conducted during the light phase (10:00–17:00 h). Antagonism studies have been performed using naloxone, the serotonergic receptor antagonist cyproheptadine, the histamine H1-receptor antagonist chlorpheniramineand the histamine H2-receptor antagonist cimetidine. Indomethacin has been used as References drug. No conflict of interest statement

Studies characteristics in response to PICOS (participants/population, interventions, comparisons, outcomes, and study design) question for records including acute nociceptive pain models; *n* = number of animals. The order of references in the table follows that in the text.

**TABLE 2:** Main characteristics of the studies included showing efficacy of EOs in neuropathic models.

Study	EO	Route of administration	Control	Mice strain	Pain model	Analgesic outcome and sample size	Design
Hamamura et al. (2020)	Bergamot (citrus bergamia risso) [d-limonene (39.60%), linalyl acetate (31.09%), and linalool (9.55%)]	s.c., with the aid of an osmotic pump	Jojoba wax	ddY mice	Partial sciatic nerve ligation	Reduction of the induced increase of planar activity during the light period at the 7th post-operative day (control *n* = 6; EO n = 9)	Acclimatization to the lighting conditions for 1 week. After basal measures before surgery, the mice have been observed for 14 days. Antagonism study with naloxone hydrochloride. Declaration of no conflict of interest. Sham procedure
Komatsu et al. (2018)	Bergamot (citrus bergamia risso) [0.38% d-limonene, 70.26% linalyl acetate, 18.95% linalool, 0.62% γ-terpinene, and 0.03% of β-pinene]	s.c	Jojoba wax and saline	ddY mice	Partial sciatic nerve ligation	On post-operative day 7, the EO of bergamot (5.0, 10.0, and 20.0 μg/paw) has attenuated dose-dependently mechanical allodynia, significantly at the dose of 20.0 μg/paw (*n* = 10)	Antagonism studies with naloxone methiodide (μ-opioid receptor preferring antagonist), β-funaltrexamine hydrochloride (selective μ-opioid receptor antagonist), β-endorphin antiserum, naltrindole (non-selective δ-opioid receptor antagonist) and nor-binaltorphimine (selective κ-opioid receptor antagonist). Behavioral tests have been performed between 10:00 and 16:00 h and for 2 days before the start of the experiment for acclimatation of the mice to the testing procedures. Sham procedure. No conflict of interest statement
Kuwahata et al. (2013)	Bergamot (citrus bergamia risso) [0.38% of d-limonene, 70.26% of linalyl acetate, 18.95% of linalool, 0.62% of γ-terpinene, and 0.03% of β-pinene]	s.c	Jojoba wax + saline	ddY mice	Partial sciatic nerve ligation	Dose-dependent reduction (5, 10, 20 µg) of tactile allodynia 7 days after surgery (*n* = 10)	Behavioral experiments have been carried out from 10:00 a.m. to 6:00 p.m. and mice have been used only once. Morphine hydrochloride has been used as References drug. Sham procedure. No conflict of interest statement

Studies characteristics relative to PICOS (participants/population, interventions, comparisons, outcomes, and study design) question for retrieved records about neuropathic pain models; *n* = number of animals. The order of references in the table follows that in the text.

#### 3.2.1 Essential Oils Endowed With Efficacy in Acute Nociceptive Models

Based on the obtained results, several EOs showed analgesic activity in acute nociceptive tests like the acetic acid writhings test, hot-plate test, and the formalin test, with the latter very useful since it includes features of both peripheral and central pain. In the study by Anaya-Eugenio et al. (2016) the EO of artemisia ludoviciana Nutt (Asteraceae) exerted dose-dependent antinociceptive activity in the hot-plate and the formalin test. It was less potent than the reference drug morphine and antagonism studies have revealed that it was inhibited by the non-selective opioid receptor antagonist naloxone. Inula britannica L (Asteraceae) has shown analgesia in the acetic acid writhings test, in the formalin test, in the tail-flick, and the glutamate test (Zarei et al., 2018). This effect is reversed by naloxone and potentiated by l-arginine, therefore all the studies performed with negative and positive controls highlighted the involvement of the opioid system and NO pathway (Zarei et al., 2018).

The EO of Myrcia pubiflora DC., Myrtaceae (Andrade et al., 2012) has demonstrated analgesic efficacy in the acetic acid writhings test and the formalin test, but not in the hot plate test. From the same family, the EO of Eugenia candolleana DC (Myrtaceae) reduced the number of writhings and licking behavior in the second phase of the formalin test in a dose-dependent manner (only at the dose of 100 mg/kg in the first phase, but not the nociceptive reaction in the hot-plate test (Guimaraes et al., 2009)).

Clove bud oil (Eugenia caryophyllata, Myrtaceae) significantly reduced formalin-induced pain behavior but affected tail-flick response in a variable way (Halder et al., 2012). The study by Bae et al. (2020) considered basil for its i. p. administration and demonstrated analgesic properties linked to action on δ- and µ-opioid pathways. Moreover, it provides orofacial antinociception at high doses (Venâncio et al., 2011). Aristolochia trilobata L. demonstrated strong analgesia in the formalin test and was comparable to morphine in the acetic acid test (Quintans et al., 2017).

The EO of Croton conduplicatus Kunth (Euphorbiaceae) has shown efficacy (de Oliveira Júnior et al., 2017; de Oliveira et al., 2018): in the acetic acid test; on the formalin‐induced nociceptive behavior at all the doses and in both phases, with effect antagonized by naloxone; on nociception in term of latency time at the highest dose (50 (deOliveira et al., 2018) and 100 (de Oliveira Júnior et al., 2017) mg/kg) in the hot‐plate test. In particular, the mechanism of action of this EO has been proposed to be influenced by ATP-sensitive K+ channels, opioid and cholinergic systems (de Oliveira Júnior et al., 2017; de Oliveira et al., 2018).

Croton cordiifolius Baill (Euphorbiaceae) also had effective results in acetic acid, formalin, and glutamate but not the capsaicin test. This antinociceptive effect was independent on naloxone (Nogueira et al., 2015). Croton adamantinus Müll. Arg. showed a strongly effective comparison with morphine in reducing licking and was more efficacious than indomethacin in decreasing abdominal contortions (Ximenes et al., 2013). Of the study by Hajhashemi et al. (2009) only the experiments using the EO i. p. and on mice could be included in the analysis, showing the effectiveness of Heracleum persicum to be almost comparable to indomethacin in the reduction of the number of writhings.

In the study by Jahandar et al. (2018) only the experiments performed on mice were considered. Pycnocycla bashagardiana (Apiaceae) has not proven analgesic but anti-inflammatory properties. In another study by Ulku Karabay–Yavasoglu et al. (2006) only experiments with the formalin test in mice were considered. The EO of Satureja thymbra L (Lamiaceae) was demonstrated to have antinociceptive efficacy in both the early and late (also at a lower dose) phases of the formalin test (Ulku Karabay–Yavasoglu et al., 2006).

In the study by Katsuyama et al. (2015) the EO of bergamot (Citrus bergamia Risso) demonstrated significant dose-dependent analgesia in both phases of the formalin test, only when administered in the ipsilateral hindpaw and antagonized by naloxone hydrochloride and methiodide (not able to cross the blood brain barrier), suggesting the involvement of peripheral opioid mechanisms. This was earlier observed in the capsaicin test in which it also enhanced morphine analgesia (Sakurada et al., 2011).

Neroli (Citrus aurantium L.) significantly increases reaction time (at 40 mg/kg) in the hot-plate test and significantly decreased the number of writhings in the study by Khodabakhsh et al. (2015), with the latter effect potentiated by L-nitro arginine methyl ester (l-NAME). In the study by Khalid et al. (2011) the EO of Zingiber zerumbet (L.) Smith, dose-dependent and comparable to acetylsalicylic acid, inhibited the nociceptive response to capsaicin, acetic acid, glutamate, and phorbol 12-myristate 13-acetate (PMA). *Eucalyptus* EO has significantly reduced licking time in the second phase of the formalin test in the study by Lee et al. (2019), and this effect was mediated by the opioid system. It also reduced the number of writhings in a dose-dependent manner but did not display activity on thermal hyperalgesia (Lee et al., 2019). In the study by Lima et al. (2012) the EO of Chrysopogon zizanioides L (Roberty, Poaceae) produced antinociception similar to morphine in the acetic acid test, and this effect was partially reversed by naloxone. Moreover, it reduced the licking time in the second phase of the formalin test, but it did not demonstrate any effects in the Hargreaves’ test.

A common trait is the presence of antiinflammatory analgesia devoid of thermal anti-hyperalgesic effect. The EO of Zhumeria majdae Rech. F. and Wendelbo (Lamiaceae) has displayed dose-related antinociceptive properties in the acetic acid and in the hot-plate test (Miraghazadeh et al., 2015). Chamaecyparis obtuse has also shown analgesia in the writhings and in the formalin, but not in the hot-plate test (Park et al., 2015). Furthermore, in the study by Mishra et al. (2010) *Senecio* rufinervis D.C. (Asteraceae) produced significant and dose-dependent inhibition of writhes and thermal hyperalgesia. In the study by Sharif et al. (2020) Tanacetum balsamita (Compositae) presented an anti-hyperalgesic effect. The antinociceptive properties exerted by Xylopia laevigata (Annonaceae) in the acetic acid and in the formalin test have not proven dependency on opioid pathways (Queiroz et al., 2014). The antinociceptive effect of Bunium persicum (Boiss.) is reversed by naloxone and attenuated by chlorpheniramine and cimetidine (Zendehdel et al., 2015), thus confirming the complex neuromodulation and the involvement of histamine in nociception (Hayashi et al., 2020). The main features of the studies on EOs analgesia in nociceptive models are summarized in Table 1.

#### 3.2.2 Essential Oils Endowed With Efficacy in Neuropathic Models

Studies assessing the analgesic properties of EOs in neuropathic pain models are fundamental because these painful conditions are the most appropriate to model chronic neuropathic pain in humans. In the study by Komatsu et al. (2018) the EO of bergamot (Citrus bergamia Risso) was demonstrated to reduce partial sciatic nerve ligation (PSNL)-induced mechanical allodynia on the seventh post-operative day, in which it peaks (Kusunose et al., 2010). In the study by Kuwahata et al. (2013) the EO of bergamot increased mechanical thresholds dose-dependently and significantly at a dose of 20 μg/paw (Kuwahata et al., 2013). Moreover, this anti-allodynic effect is stronger than that of comparable doses of morphine, of which the EO of bergamot enhances the activity (Kuwahata et al., 2013), and it was reversed by naloxone methiodide, peripherally μ-opioid receptor preferring antagonist, β-funaltrexamine hydrochloride, selective μ-opioid receptor antagonist, and β-endorphin antiserum, but not by the non-selective δ-opioid receptor antagonist naltrindole and by the selective κ-opioid receptor antagonist nor-binaltorphimine. Importantly, the study by Hamamura et al. (2020) in which the EO of bergamot was administered s. c. with an osmotic pump to allow a continuous delivery devoid of smell during PSNL, demonstrated that the anti-allodynic effect of this EO is systemic and does not depend on olfactory stimulation. In this study (Hamamura et al., 2020) the increase of planar activity during the light period induced by PSNL, with the maximum effect at the seventh post-operative day and like allodynia, was shown to be abolished by continuously administered EO. This effect is antagonized by naloxone hydrochloride. Observation lasting 14 days with a theoretical duration of the osmotic pump of one week can mimic administration during chronic pain. The main features of the studies on EOs anti-allodyinic properties are summarized in Table 2.

### 3.3 Risk of Bias Assessment

The studies included in the qualitative analysis were assessed for methodological quality according to the SYRCLE’s RoB tool (Hooijmans et al., 2014) and the CAMARADES checklist (Macleod et al., 2004; Hooijmans et al., 2014; Suokas et al., 2014), based on the Cochrane RoB (Sterne et al., 2019). These items comprise all the possible forms of bias. 1) Selection bias–sequence generation (allocation sequence able to produce comparable groups). 2) Selection bias–baseline characteristics (comparable and not adjusted for confounders in the analysis). 3) Selection bias–allocation concealment (during the enrollment). 4) Performance bias–random housing and randomization during the study. 5) Performance bias–blinding of investigators during the study. 6) Detection bias–random outcome assessment. 7) Detection bias–blinding of outcome assessors. 8) Attrition bias (animals eventually excluded from outcome assessment). 9) Reporting bias–reports free of selective outcome reporting. Finally, 10) other sources of bias: lack of evidence of induced pain using the selected behavioral outcome measure before EO administration and examination (i.e., sham procedure), clear description of methods with number of animals used, attention to circadian regulation for behavioral studies, use of the same observer for behavioral tests, use of control and positive and negative control drugs, sample size calculation, statement of conflict of interest, statement of compliance with animal welfare regulations and attention to ethics.

In terms of the two items regarding selection bias, no study reported the method of allocation and, even though they conducted baseline measures, none of the studies describe how experimental groups were composed to ensure homogeneity and consistency. Only the study by *Lima* and collaborators (Lima et al., 2012) in which mice with baseline latencies of more than 10 s, and studies by de Oliveira Júnior and colleagues (de Oliveira Júnior et al., 2017; de Oliveira et al., 2018) of more than 20 s, at the hot-plate were excluded from the experiments.

As reported in Table 1, five studies (Guimaraes et al., 2009; Andrade et al., 2012; Lima et al., 2012; de Oliveira Júnior et al., 2017; de Oliveira et al., 2018) adopted random housing of mice. The paper by Khodabakhsh et al. reported no randomization of mice but only of rats, which are not included in this systematic review and meta-analysis (Khodabakhsh et al., 2015). Mice were tested in a randomized order in studies by Sakurada and collaborators and Katsuyama et al., 2015 (Sakurada et al., 2011; Katsuyama et al., 2015). In the study by Bae et al. (2020) mice were randomly assigned to groups. The study by Khalid et al. (2011) used a blind, randomized design. Mice were randomly assigned to groups and experiments were performed in a blind manner in the study by Quintans and coworkers (Quintans et al., 2017). Moreover, in the study by Ximenes et al. (2013), the observation was conducted by a blind observer, but the number of animals used for behavioral testing was not reported, only for histological assays. Otherwise, the number of animals per group was reported, but studies that provided a range and not an exact number were not considered eligible for quantitative analysis. Attrition and reporting biases cannot be assessed from the full text of the included studies. Importantly, sham procedure and the certainty of exact execution of the pain model is present only in studies on allodynia, i.e., the studies by Hamamura et al. (2020), Komatsu et al. (2018a), and Kuwahata et al. (2013).

Attention to the circadian rhythm in behavioral testing was reported by the following studies: (Andrade et al., 2012; Quintans et al., 2017; Guimaraes et al., 2009; Halder et al., 2012; Katsuyama et al., 2015; Khodabakhsh et al., 2015; Miraghazadeh et al., 2015; Sakurada et al., 2011; Sharif et al., 2020; Zarei et al., 2018; Zendehdel et al., 2015; Komatsu et al., 2018b; Kuwahata et al., 2013). All the studies used control and positive and negative modulators. Importantly, multiple controls were used in the following studies (Katsuyama et al., 2015; Komatsu et al., 2018b; Kuwahata et al., 2013; Sakurada et al., 2011). Behavioral testing was conducted by the same observers in the following studies (de Oliveira Júnior et al., 2017; de Oliveira et al., 2018; Venãncio et al., 2011). Sample size calculation was not reported and the conflict of interest statement is present only in eight studies (Queiroz et al., 2014; Nogueira et al., 2015; de Oliveira Júnior et al., 2017; de Oliveira et al., 2018; Jahandar et al., 2018; Zarei et al., 2018; Lee et al., 2019; Hamamura et al., 2020). This could be due to the lack of requirement of these aspects in journals in the last few years. A statement of compliance with animal welfare regulations is reported in all the studies since it is an inclusion criterion. Moreover, six studies (Ulku Karabay–Yavasoglu et al., 2006; Venâncio et al., 2011; Queiroz et al., 2014; Khodabakhsh et al., 2015; Miraghazadeh et al., 2015; Sharif et al., 2020) also stated that they used each mouse only once, thus proving particular attention to animal welfare. Importantly, only the study by Hamamura et al. (2020) reported acclimatization to lighting conditions for one week and that an observation period of 14 days can model examination during chronic pain.

### 3.4 Meta-Analysis

**FIGURE 3:**
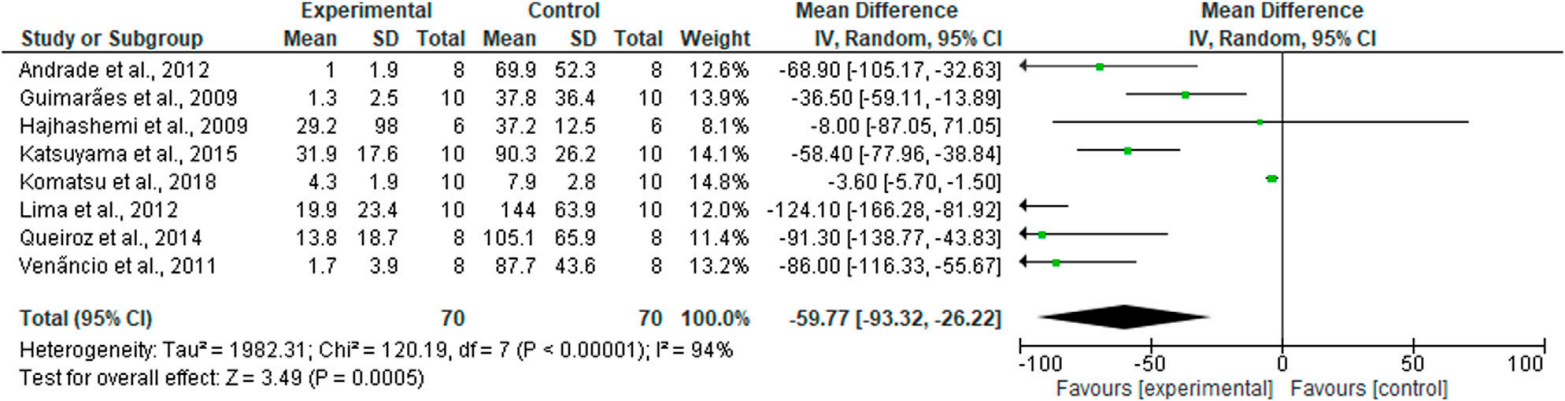
Forest plot for EOs-induced analgesia. The results of the meta-analysis favor the efficacy of the EOs, but they are affected by high heterogeneity (Mean difference MD −59.77; 95% CI (−93.32) - (−26.22); I^2^ = 94%; *p* < 0.00001).

This meta-analysis comprises eight studies for a total of 140 mice. The studies were considered comparable when the analgesic outcome was expressed as mean ± standard error of the mean (SEM) since these measures could be converted for meta-analysis in mean and standard deviation (SD). Moreover, only studies reporting the exact number of animals per group were included in quantitative analysis. Studies investigating the same pain model were considered. The formalin test pain model was chosen since it provides a biphasic nociceptive response. Due to the sensitization processes occurring during the second phase, the study on mechanical allodynia expressed has been included (Komatsu et al., 2018b). The results of the forest plot favor the analgesic efficacy of EO (Mean difference MD −59.77; 95% CI (−93.32) - (−26.22); I^2^ = 94%; *p* < 0.00001; Figure 3), but need to be carefully examined because of the extremely high heterogeneity, which is also confirmed by the asymmetry of the funnel plot analysis standing for high risk of publication bias, small studies and high differences in study precision.

## 4 Discussion

Interest in the use of EOs and aromatherapy has been continuously growing during the last few decades in parallel with preclinical research. However, in spite of all this effort of preclinical research, it is necessary to establish whether there is a strong rationale for the clinical use of EOs. This issue is even more controversial in the field of pain relief since the use of aromatherapy could reduce the dose of painkillers endowed with serious side effects, particularly in under studied areas of neuropathic pain, like opioids (Morrone et al., 2017; Scuteri et al., 2020b). Alternative pain treatments could increase time in treatment before the loss of efficacy. This is relevant to fragile populations, e.g., patients suffering from dementia, who are often undertreated compared to cognitively intact counterparts, more so during the Sars-CoV2 pandemic (Scuteri et al., 2020c).

This systematic review and meta-analysis assesses the efficacy of EOs in preclinical models of acute inflammatory nociception and neuropathic pain to understand if there is a rational basis for clinical translation. Several EOs from multiple families were found to be efficacious, in particular, croton and bergamot EOs have been extensively studied. It is noteworthy that 27 out of the 30 studies included in the qualitative analysis were only performed on acute pain models like writhings and the hot plate test. These tests are very useful since they are easy to conduct and provide fast results, but they do not resemble clinical pain conditions. Taking this into account, the quantitative analysis only includes studies on formalin test, which is more similar to clinical conditions due to its biphasic nature, and the only study on mechanical allodynia that could compare to the other seven included.

All these studies included in this review have a different experimental design and most of them present serious concerns in terms of selection, performance, and detection biases. Most studies do not adhere to the guidelines for Animal Research: Reporting *In Vivo* Experiments (ARRIVE), which are fundamental for accurate *in vivo* preclinical research (Rice et al., 2013). Another methodological aspect responsible for bias in the meta-analysis is that control groups were often used in more than one experiment, and studies including multiple comparisons can introduce errors. Thus, this systematic review and meta-analysis points to the importance of appropriate *in vivo* modeling to enhance the translational impact of pain research. Future research is necessary to improve the methodological quality and homogeneity of studies.

The results of the meta-analysis highlighted the efficacy of EOs in preclinical pain, but these data are downgraded due to the high heterogeneity of the studies. In particular, the analyzed EOs present the analgesic efficacy required by the recommendations of the Initiative on Methods, Measurement, and Pain Assessment in Clinical Trials (IMMPACT) (Turk et al., 2003), according to which a decrease in pain is defined as clinically meaningful if it accounts for a 30% to 36% reduction. However, this is referred to chronic pain and this systematic review and meta-analysis have found that only the EO of bergamot had proven efficacy both in nociceptive and in neuropathic pain models. Moreover, it was also studied for 14 days, an experimental setting suitable for modeling chronic pain (Hamamura et al., 2020).

Another important issue is that the consolidated data come from hypothesis-generating completely original preclinical studies and that they are then confirmed by hypothesis-driven studies (Mikolajewicz and Komarova, 2019). In this case, the EO of bergamot was confirmed to have strong analgesic properties in some of the most used and reliable models of inflammatory pain, i.e., formalin and capsaicin test in different experiments, sharing with most EO mechanisms involving opioid neurotransmission, and also in the PSNL. To the best of our knowledge, this is the first meta-analysis of preclinical studies on the analgesic effects of EOs and its working hypothesis was verified for bergamot EO, which could represent an important pharmacological tool for pain management in clinical settings. Along with clinical translations, more efforts are required to standardize *in vivo* preclinical studies in the field of pain research to allow for consistent research able to elucidate the mechanisms responsible for the analgesic properties of EOs.

## Data Availability

The original contributions presented in the study are included in the article.
